# Modification of the Release of Poorly Soluble Sulindac with the APTES-Modified SBA-15 Mesoporous Silica

**DOI:** 10.3390/pharmaceutics13101693

**Published:** 2021-10-15

**Authors:** Adrianna Dadej, Aneta Woźniak-Braszak, Paweł Bilski, Hanna Piotrowska-Kempisty, Małgorzata Józkowiak, Małgorzata Geszke-Moritz, Michał Moritz, Daniela Dadej, Anna Jelińska

**Affiliations:** 1 Department of Pharmaceutical Chemistry, Faculty of Pharmacy, Poznan University of Medical Sciences, Grunwaldzka 6, 60-780 Poznań, Poland; ajelinsk@ump.edu.pl; 2 Functional Materials Physics Division, Faculty of Physics, Adam Mickiewicz University, Uniwersytetu Poznańskiego 2, 61-614 Poznań, Poland; abraszak@amu.edu.pl; 3 Medical Physics and Radiospectroscopy Division, Faculty of Physics, Adam Mickiewicz University, Uniwersytetu Poznańskiego 2, 61-614 Poznań, Poland; bilski@amu.edu.pl; 4 Frank Laboratory of Neutron Physics, Joint Institute for Nuclear Research, 141980 Dubna, Russia; 5 Department of Toxicology, Faculty of Pharmacy, Poznan University of Medical Sciences, Dojazd 30, 60-631 Poznań, Poland; hpiotrow@ump.edu.pl (H.P.-K.); malgorzata.jozkowiak@gmail.com (M.J.); 6 Medical Biotechnology and Laboratory Medicine, Department of Pharmacognosy and Natural Medicines, Faculty of Pharmacy, Pomeranian Medical University in Szczecin, Al. Powstańców Wielkopolskich 72, 70-111 Szczecin, Poland; mgeszke@pum.edu.pl; 7 Medical Biotechnology and Laboratory Medicine, Department of Pharmaceutical Chemistry, Faculty of Pharmacy, Pomeranian Medical University in Szczecin, Al. Powstańców Wielkopolskich 72, 70-111 Szczecin, Poland; mmoritz@pum.edu.pl; 8 Chair and Department of Endocrinology, Metabolism and Internal Diseases, Faculty of Medicine, Poznan University of Medical Sciences, Przybyszewskiego 49, 60-355 Poznań, Poland; daniela.dadej@student.ump.edu.pl

**Keywords:** mesoporous silica, SBA-15, dissolution rate, drug delivery system, sulindac, physicochemical techniques

## Abstract

The effectiveness of oral drug administration is related to the solubility of a drug in the gastrointestinal tract and its ability to penetrate the biological membranes. As most new drugs are poorly soluble in water, there is a need to develop novel drug carriers that improve the dissolution rate and increase bioavailability. The aim of this study was to analyze the modification of sulindac release profiles in various pH levels with two APTES ((3-aminopropyl)triethoxysilane)-modified SBA-15 (Santa Barbara Amorphous-15) silicas differing in 3-aminopropyl group content. Furthermore, we investigated the cytotoxicity of the analyzed molecules. The materials were characterized by differential scanning calorimetry, powder X-ray diffraction, scanning and transmission electron microscopy, proton nuclear magnetic resonance and Fourier transformed infrared spectroscopy. Sulindac loaded on the SBA-15 was released in the hydrochloric acidic medium (pH 1.2) and phosphate buffers (pH 5.8, 6.8, and 7.4). The cytotoxicity studies were performed on Caco-2 cell line. The APTES-modified SBA-15 with a lower adsorption capacity towards sulindac released the drug in a less favorable manner. However, both analyzed materials improved the dissolution rate in acidic pH, as compared to crystalline sulindac. Moreover, the SBA-15, both before and after drug adsorption, exhibited insignificant cytotoxicity towards Caco-2 cells. The presented study evidenced that SBA-15 could serve as a non-toxic drug delivery system that enhances the dissolution rate of sulindac and improves its bioavailability.

## 1. Introduction

Since 2017, the Food and Drug Administration (FDA) has noted a growing trend in the number of new drugs approved. In 2017–2020, 46, 59, 48, and 53 new active substances were approved as medicines each year, respectively [1]. Most of the newly approved drugs exhibit poor water solubility and, therefore, a low bioavailability. A favorable form of drug delivery is the oral route due to the low cost and simplicity of use for the patient. The oral route ensures the highest rate of patient compliance and, therefore, therapy effectiveness. The efficiency of oral therapy is closely related to drug solubility in the gastrointestinal tract and the ability to penetrate the biological membranes. These two parameters determine the classification of medicinal substances into BCS (Biopharmaceutics Classification System) classes. Currently, an increasing proportion of new drugs belong to the class of water-insoluble substances, potentially leading to failures in oral therapy [2]. Therefore, simultaneously with the search for new medicinal substances, it is necessary to investigate new possibilities to improve drug dissolution. Until now, many different techniques have been proposed to improve the dissolution rate of non-steroidal anti-inflammatory drugs. As an example, the dissolution rate of poorly soluble drugs could be enhanced by supercritical carbon dioxide-based techniques, such as a supercritical antisolvent [3], a rapid expansion of supercritical solutions [4], and supercritical adsorption [5].

A promising way to improve solubility is the use of mesoporous silica materials as drug carriers. They were first presented in 1992 by Mobil Oil. The obtained materials belonged to the M41S family [6]. The synthesis of M41S materials is based on the interaction between positively charged surfactants in the presence of a negatively charged silica compound. The residual surfactants are removed by calcination or extraction [6]. The obtained materials are characterized by having an ordered and homogeneous (in size) pore network, a high pore volume, and a large specific surface area. The above properties allow for adsorption and release of drugs. The surface containing silanol groups can be functionalized, which enhances the loading and release profiles [7]. Worth noting is the fact that the analyzed materials are also characterized by high biocompatibility [2]. The first synthesized material was MCM-41 (Mobil Composition of Matter No. 41) [7].

Subsequent modifications of the synthesis process led to the discovery of the SBA-15 (Santa Barbara Amorphous-15) material, belonging to the new class of mesoporous silicas. This material is also characterized by a high degree of order and a hexagonal, two-dimensional structure, which uniquely ensure a high thermal and mechanical stability [8]. The specific surface area is further enlarged due to the presence of additional micropores in the silica walls [9]. Importantly, the SBA-15 material exhibits high biocompatibility and low toxicity [10]. Additionally, it also stabilizes the amorphous state of the adsorbed molecules by preventing their crystallization, which improves their dissolution rate [11].

Sulindac (SUL) is a class II drug of the BCS classification system that is characterized by low solubility in water. Sulindac is a non-steroidal, anti-inflammatory drug indicated in the treatment of rheumatoid arthritis, osteitis, ankylosing spondylitis, acute gouty arthritis, and other joint pain conditions (e.g., supraspinatus and acute bursitis) [12]. Moreover, sulindac has chemopreventive properties. It has been confirmed to be effective in reducing the size of colorectal adenomas in patients with Lynch syndrome and reducing the number of aberrant crypt foci [13]. Sulindac has also been proposed in the treatment of cystic fibrosis as a factor in preventing the progressive degradation of lung tissue in a mechanism independent of cyclooxygenase [14].

Until now, improvement of the dissolution rate of sulindac was achieved after the formation of complexes with cyclodextrins [15], solid dispersions with polyvinylpyrrolidone [16] or by layered double hydroxide nanomatrix formulation systems [17]. The influence of sulindac adsorption on mesoporous silicas on its dissolution rate has not to date been analyzed.

Our previous studies confirmed the ability of an SBA-15-modified material to adsorb sulindac [18]. The aim of this study was to analyze the modification of sulindac release profiles from SBA-15-functionalized silicas. Additionally, we characterized the obtained silicas before and after adsorption of sulindac by differential scanning calorimetry (DSC), X-ray diffraction (XRD), ^1^H-NMR, Fourier-transform infrared spectroscopy (FTIR), and scanning and transmission electron microscopy (SEM and TEM, respectively). Furthermore, we investigated the cytotoxicity of the analyzed molecules.

## 2. Materials and Methods

### 2.1. Chemicals and Materials

Tetraethyl orthosilicate (TEOS) (≥99.0%), hydrochloric acid (purum p.a. ≥32.0%), Pluronic P-123, (3-aminopropyl) triethoxysilane (APTES) (99%), sodium dodecyl sulfate (≥99.0%), anhydrous toluene (99.8%), and sulindac (≥98.0%) were supplied by Merck Life Science Sp.z.o.o., an affiliate of Merck KGaA, Darmstadt, Germany (Poznań, Poland). Chloroform (p.a. ≥98.5%), sodium chloride (pure p.a.), dipotassium phosphate (pure p.a.), and sodium hydroxide 0.1 mol/L (0.1 N) were purchased from Avantor Performance Materials Poland (Gliwice, Poland).

#### 2.1.1. Synthesis and Functionalization of Mesoporous Materials

We obtained the SBA-15 materials in accordance with the previously presented report [18]. We adapted the methodology presented by Zhao with modifications [8]. Briefly, we carried out the synthesis at 35 °C, and 24.0 g of poly(ethylene glycol) and poly(propylene glycol) block copolymer (Pluronic P123) (Merck Life Science Sp.z.o.o., an affiliate of Merck KGaA, Darmstadt, Germany, Poznań, Poland) was dissolved in 900 mL of aqueous HCl (1.6 M). Afterwards, we added 51.0 g of tetraethyl orthosilicate (TEOS) into the solution and magnetically stirred for 20 h. Next, the mixture was aged at 110 °C (24 h). Further, we filtered the suspension and washed using distilled water. Eventually, the precipitate was air dried, and the final product was calcined at 500 °C (6 h with heating rate 1 °C/min). We obtained the APTES ((3-aminopropyl) triethoxysilane) modified SBA-15 silica (Merck Life Science Sp.z.o.o., an affiliate of Merck KGaA, Darmstadt, Germany, Poznań, Poland) by grafting method in accordance with methodology presented by Geszke-Moritz and Moritz [19]. Using different molar ratios (0.05 and 0.20) of the trialkoxysilane to the SBA-15 silica, two different functionalized materials were obtained (SBA-15-A_0.05_ and SBA-15-A_0.20_, respectively).

#### 2.1.2. Sulindac Adsorption Studies

SBA-15 material modified with 3-aminopropyl groups was used for the tests. Adsorption of sulindac was carried out at 25 °C in 2-propanol (Avantor Performance Materials Poland, Gliwice, Poland). To obtain each sample, we used 5 mL of 3.0 mg/mL SUL solution and 50.0 mg of SBA-15-A_0.05_ or SBA-15-A_0.20_. After stirring the mixture for 48 h, the suspension was centrifuged at 6000 rpm for 15 min. The precipitate was air dried. The obtained material was designated as SBA-15-A_0.05_:SUL and SBA-15-A_0.20_:SUL [18].

The amount of adsorbed SUL was calculated as the difference in the concentrations of the solutions before and after adsorption using a UV/VIS LAMBDA 20 Perkin Elmer spectrophotometer (PerkinElmer, Inc., Waltham, MA, USA) at 396 nm. The amount of SUL adsorbed in SBA-15-A_0.05_ and SBA-15-A_0.20_ was 90.5 mg/g and 221.3 mg/g, respectively [18].

### 2.2. Characterization Methods

#### 2.2.1. Powder X-ray Diffraction (XRD)

The small angle XRD patterns were collected with Bruker D8 Advance (Billerica, MA, USA) in the range of 0.6 < 2θ < 8.0°. The wide-angle XRD patterns were acquired with Bruker D2 Phaser (Billerica, MA, USA) in the range of 5.0 < 2θ < 45.0°. The measurements were performed with a 0.02° step width associated with a step time of 1–2 s.

#### 2.2.2. Differential Scanning Calorimetry (DSC)

DSC thermograms were obtained using DSC 214 Polyma Netzsch (Netzsch Group, Selb, Germany) in a nitrogen atmosphere (30 mL/min). The samples were heated up to 250 °C with a scanning rate of 5 °C/min. The weight of each sample was approximately 5.0 mg.

#### 2.2.3. Transmission and Scanning Electron Microscopy (TEM and SEM)

TEM images were obtained by JOEL JEM 1200 EX electron microscope (JEOL Ltd., Tokyo, Japan) operated at 80 kV. SEM images were taken on Zeiss EVO-40 electron microscope (Carl Zeiss AG, Oberkochen, Germany).

#### 2.2.4. Fourier Transformed Infrared Spectroscopy (FTIR)

The FTIR analyses were performed on Bruker FTIR IFS 66/s spectrometer (Billerica, MA, USA) using the KBr pellet technique in the wavenumber range 400–4000 cm^−1^ with the resolution of 1 cm^−1^. Tablets were formulated by mixing 1 mg of the studied substance with 200 mg of potassium bromide until a homogeneous mixture was obtained.

#### 2.2.5. Proton Nuclear Magnetic Resonance (^1^H-NMR)

The proton spin-lattice relaxation times *T*_1_ in the laboratory frame were measured on a pulse spectrometer operating at a frequency of 25 MHz (El-Lab Tel-Atomic, Jackson, MI, USA) using the standard saturation recovery sequence ending with a solid echo [20,21,22].

All measurements were carried out with 3% error. The samples of SUL, SBA-15-A_0.05_:SUL, and SBA-15-A_0.20_:SUL were sealed in glass tubes and degassed in order to remove humidity effects and paramagnetic oxygen. Measurements were performed in a wide temperature range from 80 K to 300 K. The temperature of the sample was stabilized within 15 min and controlled using a gas-flow cryostat and monitored with a Pt resistor to an accuracy of 0.5 K. Due to a weak signal in the SBA-15-A_0.05_:SUL and SBA-15-A_0.20_:SUL samples that was related to a small number of protons, multiple-signal accumulation was applied. The *T*_1_ values were extracted by fitting the magnetization recovery to the one or be-exponential function for SUL, and SBA-15-A_0.05_:SUL and SBA-15-A_0.20_:SUL samples, respectively.

#### 2.2.6. Spectrophotometry

UV/VIS analyses were performed using UV/VIS LAMBDA 20 Perkin Elmer spectrophotometer (PerkinElmer, Inc., Waltham, MA, USA).

### 2.3. Drug Release Studies

The release studies of sulindac from mesoporous silicas and the dissolution tests of pure sulindac were carried out on an Electrolab EDT 08Lx apparatus (Electrolab, Janki Impex, Gujarat, India) using the rotating paddle method at 37 ± 0.5 °C. Samples corresponding to 4 mg of sulindac were used for each analysis. The release volume was reduced to 500 mL under sink conditions, and the rotation speed was set at 70 rpm. During the test, the following media were used: a hydrochloric acidic medium at pH = 1.2 ± 0.1 and phosphate buffers at pH = 5.8 ± 0.1, 6.8 ± 0.1, and 7.4 ± 0.1 (European Pharmacopoeia 10th Edition). After the specified time (5, 15, 30, 45, 60, 90, and 120 min), 4 mL of liquid was collected and then filtered through a 0.22 µm PTFE filter (Bionovo, Legnica, Poland), and the sulindac concentration was assessed by UV spectrophotometry at 333 nm for the hydrochloric acidic medium and 327 nm for the phosphate buffers. The removed volume of dissolution fluid was immediately replaced by 4 mL of fresh medium (37 ± 0.5 °C). All samples were analyzed in triplicate.

A method based on the calculation of the similarity index *f*_2_ was used to compare the release profiles. The similarity coefficients *f*_2_ were calculated according to the formula below [23]:

(1) $$f_{2}=50\times \log_{\;}\{[1+\frac{1}{n}\sum_{t=1}^{n}\left(R_{t}-T_{t}\right){}^{2}]{}^{-0.5}\times 100\}$$

where *n* is number of sample points, *R_t_* and *T_t_* are the percentage of dissolved/released SUL from the reference and test samples, respectively, at time *t.*

### 2.4. Cytotoxicity Studies

#### 2.4.1. Cell Culture

Caco-2 human colon adenocarcinoma cell line was supplied from the European Collection of Authenticated Cell Cultures ECACC 86010202 (Merck Life Science Sp.z.o.o., an affiliate of Merck KGaA, Darmstadt, Germany, Poznań, Poland). Caco-2 cells were maintained in phenol-free DMEM medium (Merck Life Science Sp.z.o.o., an affiliate of Merck KGaA, Darmstadt, Germany, Poznań, Poland), supplemented with 10–20% fetal bovine serum (FBS), 1% non-essential amino acids mixture, 2 mM glutamine, streptomycin (0.1 mg/mL), and penicillin (100 U/mL) (Merck Life Science Sp.z.o.o., an affiliate of Merck KGaA, Darmstadt, Germany, Poznań, Poland). The tests were carried out on Caco-2 cells from 20 to X passage.

#### 2.4.2. Cell Viability

Caco-2 cells were cultivated under the standard conditions in a humidified atmosphere containing 95% air and 5% CO_2_ at 37 °C. To evaluate the effects of SUL, SBA-15-A_0.20_, and SBA-15-A_0.20_:SUL on cell viability, confluent stock cultures were detached using trypsin and seeded in 96-well plates at a density of 2 × 10^4^ cells/well in 150 µL of growth medium. After attachment, the cells were left for 48 h and analyzed compounds were added. We analyzed the concentrations of SBA-15-A_0.20_ of 0.125–1.0 mg/ mL and the SUL concentrations, which corresponded to the amount of the SUL adsorbed on the SBA-15-A_0.20_ used for the tests. We measured the cell viability after 2 h of incubation using CellTiter-Glo Luminescent Cell Viability Assay (Promega, Madison, WI, USA), in accordance with the manufacturer’s instructions.

## 3. Results and Discussion

### 3.1. Powder X-ray Diffraction (XRD)

**Figure 1:**
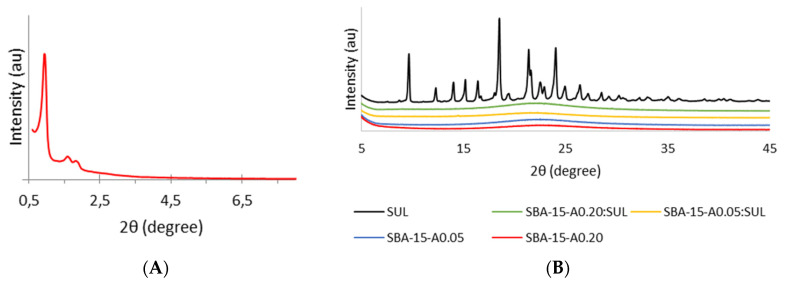
(**A**) Small-angle XRD pattern of the SBA-15-A_0.20_; (**B**) Wide-angle XRD patterns of SUL, SBA-15-A_0.05_:SUL, SBA-15-A_0.20_:SUL, SBA-15-A_0.05_, and SBA-15-A_0.20_.

The XRD analysis allowed us to distinguish between the crystalline and amorphous nature of the samples. Figure 1A presents the small-angle XRD profile of SBA-15-A_0.20_. The diffractogram reveals well-resolved peaks that correspond to (100), (110), and (200) planes and prove an ordered structure and hexagonal symmetry with the space group p6mm of analyzed silica [8].

Figure 1B shows the wide-angle diffractogram profiles of SUL, SBA-15-A_0.05,_ SBA-15-A_0.20_, SBA-15-A_0.05_:SUL, and SBA-15-A_0.20_:SUL. The diffractogram of SUL reveals the crystalline state of the substance, as it shows typical peaks at diffraction angles (2θ) of 9.67°, 12.31°, 14.04°, 15.02°, 16.42°, 18.53°, 21.4°, 22.52°, 24.06°, 24.95°, 26.44°, 27.23°, and 28.55° [24]. The XRD patterns for both APTES-modified silicas present one wide peak with the maximum located at ≈22.5°, corroborating the amorphous nature of the materials. Similarly, for samples with adsorbed SUL, we observe only one peak characteristic for the carrier. Those diffractograms show no peaks typical for crystalline sulindac, which proves that the adsorbed drug is in an amorphous state. Previous studies have also reported the transition of the drug from crystalline to amorphous state during the adsorption process on SBA-15 [25].

### 3.2. Differential Scanning Calorimetry (DSC)

**Figure 2:**
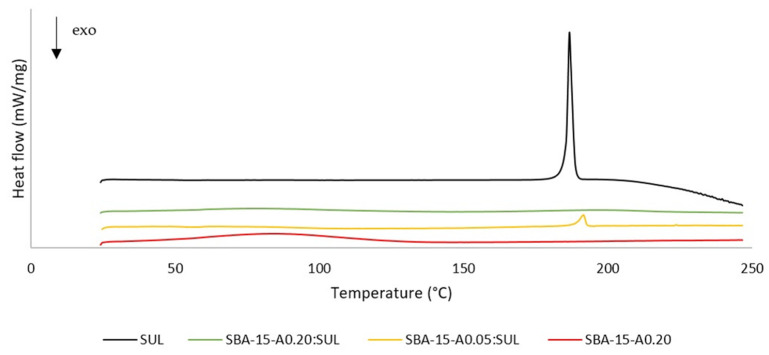
DSC thermograms of SUL, SBA-15-A_0.05_:SUL, SBA-15-A_0.20_:SUL, and SBA-15-A_0.20_.

We applied the DSC method in order to analyze the thermal properties of samples and to evaluate their physical state. DSC allows the determination of glass transitions and the investigation of the crystallization and melting behavior of the analyzed materials. The DSC curves for SUL, SBA-15-A_0.20_, SBA-15-A_0.05_:SUL, and SBA-15-A_0.20_:SUL are presented in Figure 2. The DSC curve for SUL presents one endothermic peak at 186.6 °C, which is assigned to the melting temperature of the substance. Above the temperature of 205 °C, an exothermic peak begins, indicating the decomposition of the drug [26].

The DSC curve for SBA-15-A_0.20_ shows a peak correlated with glass transition at 65 °C. The presence of this peak proves the amorphous state of the carrier. In the analyzed temperature range, the SBA-15-A_0.20_ is thermally stable as there are no melting or degradation peaks [27].

Likewise, the DSC curve for SBA-15-A_0.20_:SUL presents no endothermic or exothermic peaks, which serves to corroborate the amorphous state of the SUL adsorbed inside the mesopores of the SBA-15-A_0.20_ [28].

For SBA-15-A_0.05_:SUL, we obtained the curve with one endothermic peak at 191.6 °C. The presence of this peak suggests that the crystalline SUL has partially adsorbed on the surface of SBA-15-A_0.05_ [28]. However, our XRD study showed conflicting results.

### 3.3. Transmission and Scanning Electron Microscopy (TEM and SEM)

**Figure 3:**
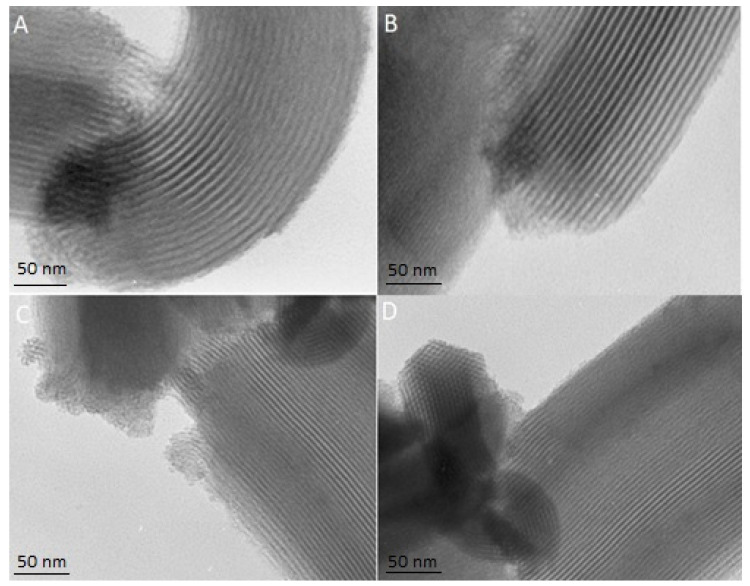
TEM images of (**A**) SBA-15-A_0.05_:SUL, (**B**) SBA-15-A_0.20_:SUL, (**C**) SBA-15-A_0.05,_ and (**D**) SBA-15-A_0.20_.

The TEM and SEM micrographs enable the evaluation of the structure and the morphology of mesoporous materials. The TEM images present the structure of the SBA-15-A_0.05_:SUL, SBA-15-A_0.20_:SUL, SBA-15-A_0.05_, and SBA-15-A_0.20_ (Figure 3). The TEM micrographs show the parallel mesoporous channels, indicating the two-dimensional structure of the mesoporous silica. Noteworthy, too, is that the structure of SBA-15-A_0.20_ and of SBA-15-A_0.05_ remains unchanged after SUL adsorption. Similar results were reported in the literature data [29,30].

**Figure 4:**
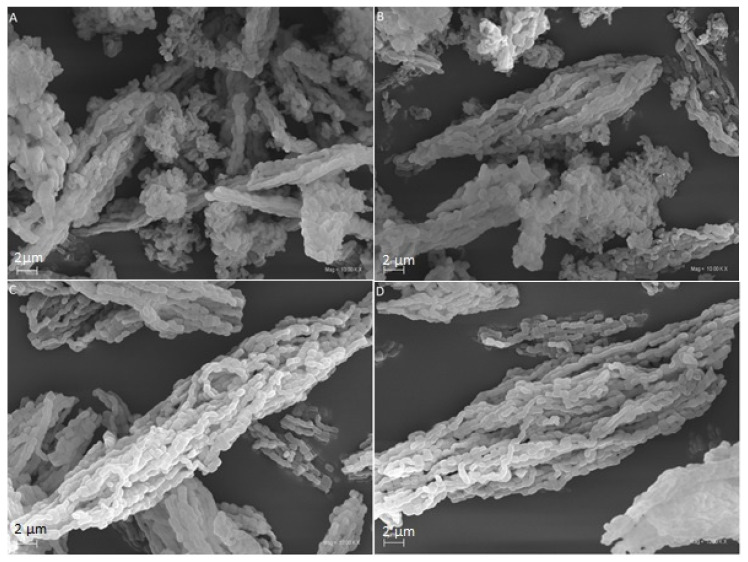
SEM images of (**A**) SBA-15-A_0.05_:SUL, (**B**) SBA-15-A_0.20_:SUL, (**C**) SBA-15-A_0.05_, and (**D**) SBA-15-A_0.20_.

All SEM images (Figure 4) present well-formed, rod-like particles of about 1 µm in length, which aggregated into larger linear, chain-like structures. The SEM micrographs of the samples after incorporation present no sulindac crystals, which indicate the adsorption of the sulindac inside the mesopores. Our observations are consistent with the results of Letchmanan et al. [29].

### 3.4. Fourier Transformed Infrared Spectroscopy (FTIR)

**Figure 5:**
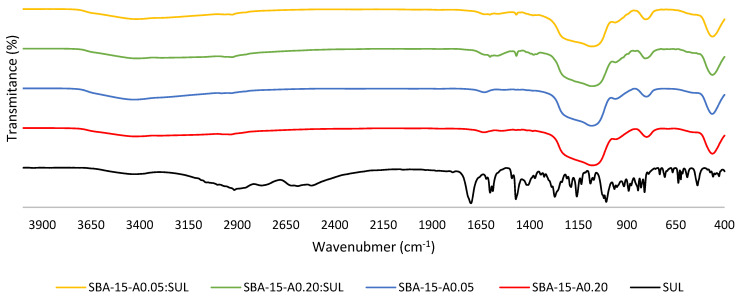
FT-IR spectra of SUL, SBA-15-A_0.05_, SBA-15-A_0.20_, SBA-15-A_0.05_:SUL, and SBA-15-A_0.20_:SUL.

The FT-IR spectroscopy allowed the determination of the functional groups in the sample and the identification of the chemical bonds occurring in the molecule. Figure 5 presents the FT-IR spectra of SUL, APTES-modified SBA-15, and its SUL-loaded forms.

Our results are in line with the previously reported data. In the spectra of the APTES-modified SBA-15, the bands around 3430 cm^−1^ and 1630 cm^−1^ are identified as the stretching and bending vibrations of the OH groups (Si–OH), respectively [31]. The broad band in the range 1200–1080 cm^−1^ can be assigned to the Si–O–Si asymmetric stretching vibrations. The symmetric stretching and bending vibrations of Si–O–Si are located at 800 cm^−1^ and 460 cm^−1^, respectively [31,32]. The band around 2920 cm^−1^ is attributed to stretching vibrations from the propyl chain and the band at 1550 cm^−1^ is associated to the N–H bending vibrations. The presence of those bands confirms the functionalization of SBA-15 silica [33].

In the spectrum of SUL, the band at 3064 cm^−1^ is related to the C–H fundamental stretching vibrations of the aromatic ring. The bands at 2994 cm^−1^ and 2911 cm^−1^ are assigned to the asymmetric stretching vibrations of CH_3_ and CH_2_ groups, respectively. The peaks at 2768 cm^−1^, 2582 cm^−1,^ and 2512 cm^−1^ correspond to the stretching vibrations of the OH group. The band at 1701 cm^−1^ is associated with the stretching vibrations of the C=O group. The stretching vibrations of the C–C bonds in the aromatic ring appear at 1602 cm^−1^, 1588 cm^−1^, and 1469 cm^−1^. The peak identified at 1155 cm^−1^ is related to the stretching vibrations of C–F. The S–O bonds can be localized at 1010 cm^−1^ and 1005 cm^−1^ [26].

The comparison of FT-IR spectra of SUL, SBA-15-A_0.05_, and SBA-15-A_0.20_ with its loaded forms proves the presence of the characteristic peak at 1469 cm^−1^ originating from SUL on the SBA-15-A_0.05_:SUL and SBA-15-A_0.20_:SUL spectra. It confirms the successful adsorption of sulindac.

### 3.5. Proton Nuclear Magnetic Resonance ^1^H-NMR

The nuclear magnetic resonance technique (NMR) is a very useful tool for studying the molecular dynamics of a drug molecule. The analysis of the temperature dependence of spin-lattice relaxation times *T*_1_ provided essential information about the physicochemical properties of the investigated substances [34,35].

**Figure 6:**
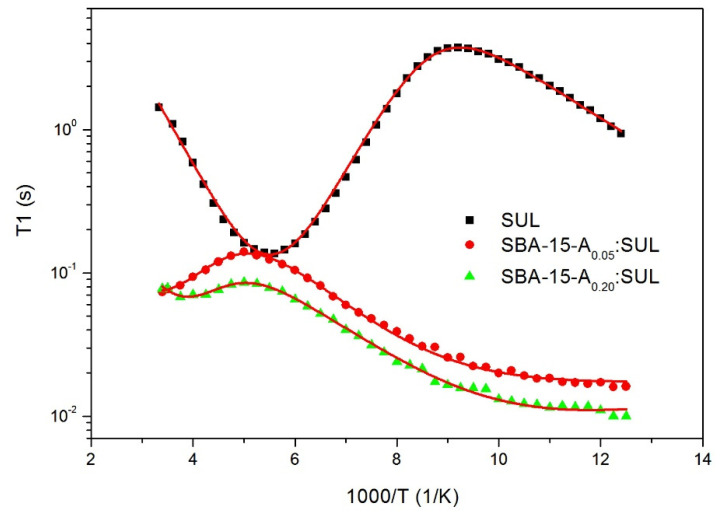
Temperature dependence of the spin-relaxation time *T*_1_ in the laboratory frame for SUL, SBA-15-A_0.05_:SUL, and SBA-15-A_0.20_:SUL.

Figure 6 presents the proton spin-lattice relaxation times *T*_1_ in the laboratory frame as a function of reciprocal temperature for SUL, SBA-15-A_0.05_:SUL, and SBA-15-A_0.20_:SUL, respectively.

For SUL, the recovery of magnetization M_z_(t) was one exponential in the entire temperature range. The spin-relaxation time *T*_1_ was estimated from fitting the following equation to the experimental data:

(2) $$M_{z}\left(t\right)=M_{0}\left(1-\exp\left(-\frac{t}{T_{1}}\right)\right)$$

where M_0_ is the equilibrium magnetization and *T*_1_ is the relaxation time.

The recovery of the magnetization for SBA-15-A_0.05_:SUL and SBA-15-A_0.20_:SUL was bi-exponential, resulting in two magnetization fractions with a ratio of 1:9.

The temperature dependence of the longer component of the relaxation times for a drug incorporated into mesoporous silica had a similar course to the relaxation times for SUL. However, due to its small contribution to and its large dispersion of the experimental data, its interpretation was rejected. The existence of this component for both samples SBA-15-A_0.05_:SUL and SBA-15-A_0.20_:SUL indicated that some of the drug was not adsorbed to the interior of the silica and remained on its surface. Based on the experimental data, it was determined that less than 10% of the drug was localized outside the pores and about 90% inside. Obtained results are consistent with our DSC analysis, which confirmed partial adsorption on the silica’s surface.

The temperature dependence of *T*_1_ relaxation times of SUL, SBA-15-A_0.05_: SUL and SBA-15-A_0.20_: SUL was analyzed, taking into account the dipole-dipole interactions modulated by molecular motions as described by Bloembergen-Purcell-Pound (BPP) theory [36]. It was assumed that the *T*_1_ values were determined by dipolar interactions modulated by two different molecular processes: the hindered rotation of methyl CH_3_ groups around the threefold axes C3 and the jumping of hydrogen atoms in hydrogen bonds at low temperature. To obtain the activation parameters of the two distinctive processes, the temperature curves *T*_1_ were determined by fitting the following Equations (3)–(5) to the experimental data [37,38]:

(3) $$\frac{1}{T_{1}}=\frac{2}{3}\gamma^{2}\sum_{k}\Delta M_{2k}\left[J_{k}\left(\omega \right)+4J_{k}\left(2\omega \right)\right]$$

where *γ* is a gyromagnetic ratio of protons, Δ*M_2k_* is a reduction of the second moment, and *J_k_(ω)* is the spectral density function for the angular frequency *ω*.

It was assumed that motions were thermally activated, and the temperature dependence of the correlation time τ_*c*_ was expressed by the Arrhenius formula:

(4) $$\tau_{c}=\tau_{0}exp\left(\frac{E_{a}}{RT}\right)$$

where τ_0_ is the pre-exponential factor corresponding to the correlation time at an infinite temperature, *E_a_* is the activation energy of molecular motion, and *R* is the universal gas constant.

For the interpretation of the CH_3_ motion, the classical BPP spectral density function was applied [39]:

(5) $$J\left(\omega \right)=\frac{2\tau_{c}}{1+\omega^{2}\tau_{c}^{2}}$$

A sharp, symmetrical minimum of 128 ms at about 182 K (−91 °C), depicted in Figure 6, was attributed to the hindered rotation of methyl CH_3_ groups around the threefold axes C3. For sulindac, the methyl group reorientation was characterized by the activation energy *E_a_* of 12.2 kJ/mol and a τ_0_ value of 1.2 × 10^−12^ s, which was confirmed by prior ^1^H-NMR results [34,40,41]. In the low-temperature region, a decrease of the relaxation times *T*_1_ was observed, and the contribution of another type of molecular motion was considered. The activation parameters of this motion are collected in Table 1. It was assumed that, at low temperatures, the relaxation was modulated by the jumping of hydrogen atoms in hydrogen bonds [42].

For the SBA-15-A_0.05_:SUL and SBA-15-A_0.20_:SUL samples, different temperature *T*_1_ curves were obtained in comparison to SUL. The minima associated with the relaxation processes for sulindac incorporated into mesoporous silica appeared at the higher temperature, which implied that the molecular dynamics were hindered for molecules of SUL inside the pores. The analysis of ^1^H-NMR data was performed according to the procedure described for SUL; however, to describe a low, wide temperature minimum of relaxation times *T*_1_, the spectral density function *J*(ω), given by Davidson and Cole, was used [43]:

(6) $$J\left(\omega ,\tau_{c},\beta \right)=\frac{2}{\omega }\left[\frac{\sin\left(\beta \arctan\left(\omega \tau_{c}\right)\right)}{{\left(1+\omega^{2}\tau_{c}^{2}\right)}^{\frac{\beta }{2}}}\right]$$

where τ_c_ is the upper cut-off correlation time and β is the distribution width of the correlation times.

The activation parameters of these molecular processes were extracted and collected in Table 1. The ^1^H-NMR results showed that the activation energies of the hindered rotation of the CH_3_ groups and of low temperature motion slightly increased for the drug adsorbed in the silica pores, as compared to SUL. In addition, a shorter relaxation time *T*_1_ for the incorporated drug in the SBA-15 mesopores confirmed that the protons of the sulindac molecules incorporated into the silica were more mobile, which gave them a greater degree of freedom. The increased mobility of these systems evidenced the transfer of the crystalline structure to the amorphous one, which could improve the bioavailability of the drug [44,45].

Comparing ^1^H-NMR data for the SBA-15-A_0.05_:SUL and SBA-15-A_0.20_:SUL samples, it can be stated that the relaxation times for the SBA-15-A_0.20_:SUL sample were the shortest, and, therefore, the mobility of protons in this system was the greatest. It can be assumed that an increase in the 3-aminopropyl groups in the silica structure may attenuate the interactions between the sulindac molecules.

### 3.6. Drug Release Studies

Sulindac belongs to BCS class II and is characterized by high permeability but low solubility, which hinders its absorption, but its absorption may be improved by increasing its dissolution rate. Sulindac is a drug of arylalkanoic acid with a pK_a_ of 4.27 [46], and as such, in the stomach where the pH is low, sulindac is poorly soluble, and its absorption is limited. In the small intestine, where the pH is higher, most of the drug molecules are ionized and partially dissolved [47].

**Figure 7:**
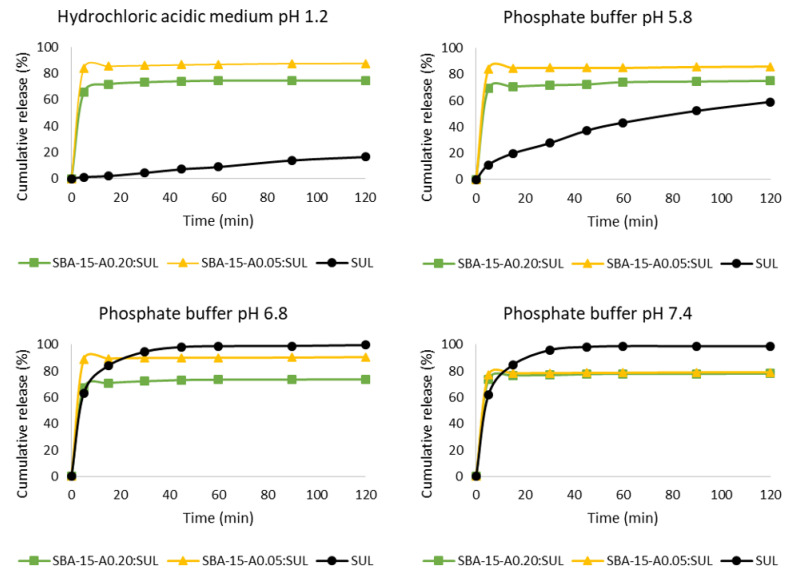
Dissolution profiles of SUL, SBA-15-A_0.20_:SUL, and SBA-15-A_0.05_:SUL.

The sulindac release profiles from SBA-15-A_0.05_:SUL and SBA-15-A_0.20_:SUL are presented in Figure 7. Our results confirmed a very low dissolution of sulindac at a low pH, mimicking the conditions in the stomach (hydrochloric acidic medium at a pH of 1.2). The increase of pH resulted in an increase of the amount of dissolved sulindac. The obtained results were consistent with the literature [46].

The dissolution tests at pH = 1.2 and pH = 5.8 proved that considerably more drug was released from SBA-15-A_0.05_:SUL and SBA-15-A_0.20_:SUL, respectively, than was dissolved from its crystalline form. In the hydrochloric acidic medium at pH = 1.2 and after 5 min of the test, 65.6% of sulindac was released from SBA-15-A_0.20_:SUL and almost 84% from SBA-15-A_0.05_:SUL. Only 1.2% of the crystalline sulindac was dissolved at the same time. After 2 h of the test, just 16.5% of the sulindac was dissolved while 72% of the drug was released from SBA-15-A_0.20_:SUL and almost 88% from SBA-15-A_0.05_:SUL. The increased dissolution rate of SUL adsorbed on the mesoporous silica was related to the amorphous state of the drug and the reduction in particle size of sulindac [29]. Substances in the amorphous form are characterized by reduced lattice energy and, therefore, higher dissolution rates and increased bioavailability. Moreover, the hydrophilic nature of the mesoporous silica surface facilitates the wetting and dispersion of the adsorbed SUL and accelerating its dissolution [10].

In a phosphate buffer at pH = 5.8, the differences in the release profiles of sulindac decreased, but remained significant. After 120 min, 59% of the crystalline form of sulindac dissolved, whereas 73.7% and almost 85% of the drug was released from SBA-15-A_0.20_:SUL and SBA-15-A_0.05_:SUL, respectively.

Based on the values of the similarity coefficient *f*_2_ (Table 2), we concluded that the observed drug release rate was higher than its dissolution rate. The improvement in the dissolution rate of sulindac may have resulted from its adsorption on mesoporous carriers in an amorphous state [31]. The amorphous state of the drug was confirmed in our DSC and XRD analyses.

At higher pHs (6.8 and 7.4), an initially greater amount of sulindac was released from the silicas than that which was dissolved from the crystalline form. However, after 30 min at pH = 6.8 and after 15 min at pH = 7.4, the amount of dissolved sulindac exceeded the amount of released substance from both silica types. After 45 min of the test, the amount of the dissolved sulindac surpassed 98%.

In contrast to the pH-dependent dissolution of sulindac, the release of sulindac from the mesoporous materials remained constant under different pH conditions. The drug release profiles from both silicas were similar, as evidenced by the *f*_2_ similarity coefficients above 50 (Table 3). The exception was the SBA-15-A_0.05_:SUL release profile in a phosphate buffer at pH = 6.8 and pH = 7.4. We observed a reduction in the amount of sulindac released from SBA-15-A_0.05_:SUL as the pH values increased.

What was noteworthy was that none of the analyzed conditions had allowed a complete release of the drug from the carriers. This could be due to the potential diffusion resistances or the sorption equilibrium of sulindac with the modified silica surfaces [48].

SBA-15-A_0.20_:SUL demonstrated a less favorable sulindac release profile at all of the studied pH values, as compared to SBA-15-A_0.05_:SUL. This may have resulted from the different pore sizes of the silicas. The larger pores in SBA-15-A_0.05_ allowed an easier diffusion of the drug molecules from the silica into the medium, resulting in a greater proportion of the released drug [32,49].

### 3.7. Cytotoxicity Studies

**Figure 8:**
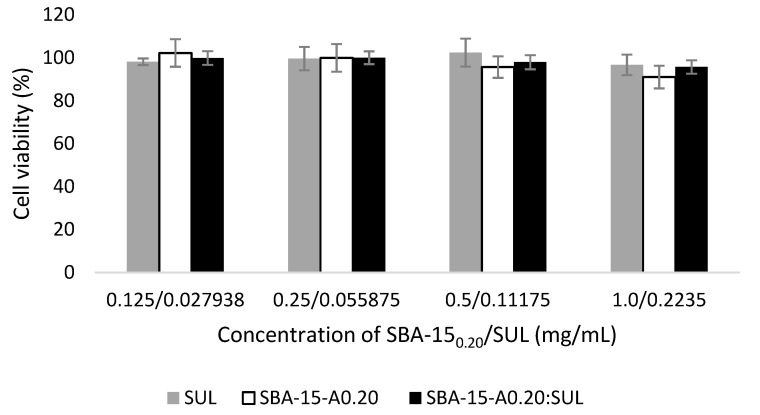
Caco-2 cell viability results after 2 h incubation with SBA-15-A_0.20_, SBA-15-A_0.20_:SUL, and SUL at 37 °C.

We assessed the biocompatibility of analyzed silica before and after sulindac loading. For this purpose, we used the cell visibility test, which assumes that the number of viable cells is proportional to the amount of ATP (adenosine triphosphate) produced by metabolically active cells [50]. To assess the cytotoxicity of analyzed samples, we used the Caco-2 cells (human colorectal adenocarcinoma cells) that resemble the human epithelial cells of the small intestine [51]. Figure 8 presents the viability of Caco-2 cells after 2 h of exposure to SBA-15-A_0.20_, SBA-15-A_0.20_:SUL (0.125, 0.25, 0.50, and 1.0 mg/mL), and SUL (0.0279, 0.0559, 0.1118, and 0.2235 mg/mL). CellTiter-Glo Luminescence assay revealed no cytotoxic effect of the analyzed samples on the -2 cells. Obtained results were in line with previous findings that SBA-15 mesoporous silica showed negligible cytotoxicity towards various types of human cell lines [28,52]. We also noted no differences between the cell viability of the samples between all analyzed concentrations. The results suggested that the adsorption of SUL to SBA-15-A_0.20_ does not influence the silica’s cytotoxicity.

## 4. Conclusions

Our study investigated the possibility of increasing the dissolution rate of the poorly soluble sulindac using adsorption on APTES-modified mesoporous silicas. Our SEM, DSC, XRD, and ^1^H-NMR analyses proved that adsorption of the drug on the analyzed silicas provided the amorphous state of the adsorbed substance in 90% of the resulting specimens. We demonstrated that APTES-modified SBA-15 containing more 3-aminopropyl substituents, which are characterized by a higher adsorption capacity towards sulindac, released the drug in a less favorable manner, as compared to SBA-15-A_0.05_. We observed a higher dissolution rate of SUL when incorporated into SBA-15-A_0.20_ and SBA-15-A_0.05_ at acidic pH, as compared to the crystalline SUL. The cytotoxicity of the analyzed carrier before and after adsorption of SUL was insignificant. The presented study evidenced that mesoporous silica SBA-15 is applicable as a non-toxic, effective drug delivery system for the poorly soluble sulindac.

## Figures and Tables

**Table 1:** Motional parameters obtained for SUL, SBA-15-A_0.05_:SUL, and SBA-15-A_0.20_:SUL. The values of uncertainty of the estimated parameters were lower than 10%.

Sample	Hindered Rotation of CH_3_ Group		Jump of Hydrogen Atom in Hydrogen Bonds		
	τ_0_ (s)	*E_a_* (kJ/mol)	τ_0_ (s)	*E_a_* (kJ/mol)	β
SUL	1.2 × 10^−12^	12.2	4.2 × 10^−13^	4.3	-
SBA-15-A_0.05_:SUL	2.2 × 10^−11^	12.7	2.1 × 10^−11^	4.7	0.2
SBA-15-A_0.20_:SUL	1.2 × 10^−11^	12.9	1.9 × 10^−11^	4.5	0.2

**Table 2:** Comparison of the similarity coefficient *f*_2_ between crystalline SUL and mesoporous silica.

	Hydrochloric Acidic Medium pH = 1.2			Phosphate Buffer pH = 5.8			Phosphate Buffer pH = 6.8			Phosphate Buffer pH = 7.4		
	SBA-15-A_0.20_:SUL	SBA-15-A_0.05_:SUL	SUL	SBA-15-A_0.20_:SUL	SBA-15-A_0.05_:SUL	SUL	SBA-15-A_0.20_:SUL	SBA-15-A_0.05_:SUL	SUL	SBA-15-A_0.20_:SUL	SBA-15-A_0.05_:SUL	SUL
SBA-15-A_0.20_:SUL	-	43.03	9.34	-	44.92	20.29	-	37.28	33.23	-	85.36	37.08
SBA-15-A_0.05_:SUL		-	5.22		-	14.38		-	45.91		-	37.81
SUL			-			-			-			-

**Table 3:** Comparison of the similarity coefficient *f*_2_ between mesoporous silicas.

	SBA-15-A_0.20_:SUL			SBA-15-A_0.05_:SUL		
	Phosphate Buffer pH = 5.8	Phosphate Buffer pH = 6.8	Phosphate Buffer pH = 7.4	Phosphate Buffer pH = 5.8	Phosphate Buffer pH = 6.8	Phosphate Buffer pH = 7.4
Hydrochloric acidic medium pH = 1.2	85.49657	91.37849	66.31096	87.36534	70.3508	55.22457
Phosphate buffer pH = 5.8	-	92.9635	66.12648	-	64.39402	59.18928
Phosphate buffer pH = 6.8		-	64.98873		-	46.85479
